# Palmar syringofibroadenoma-like lesions in Clouston syndrome treated with CO_2_ ablative laser

**DOI:** 10.1016/j.jdcr.2023.07.014

**Published:** 2023-07-26

**Authors:** Meryem Safoine, Danielle Bouffard, Elena Netchiporouk, Catherine Maari

**Affiliations:** a Division of Dermatology, Department of Medicine, Centre hospitalier de l’Université de Montréal; b Department of Pathology, Centre hospitalier de l’Université de Montréal; c Division of Dermatology, Department of Medicine, McGill University Health Centre

**Keywords:** ablative laser, Clouston syndrome, CO_2_ laser, ectodermal dysplasia, palmar keratoderma, syringofibroadenoma

## Introduction

Clouston syndrome, also known as hidrotic ectodermal dysplasia type 2, is a rare autosomal dominant genodermatosis caused by mutations in the gap junction protein beta-6 gene. It is classically characterized by a variable degree of alopecia, nail dystrophy, and progressive palmoplantar keratoderma.^1^ There are a few reports on the association between eccrine syringofibroadenoma (ESFA) and Clouston syndrome.^2^ Syringofibroadenoma is an uncommon benign adnexal tumor that originates from the acrosyringium cells of eccrine sudoriferous glands, typically occurring on the extremities of elderly patients.^3^ Herein, we describe 2 related patients, a mother and her daughter, both having Clouston syndrome and presenting with palmar syringofibroadenoma. The association between Clouston syndrome and syringofibroadenoma has been scarcely reported.^4^

Syringofibroadenoma is usually managed with procedural approaches such as surgical excision, cryotherapy, and laser, among others.^5^ However, in the case of Clouston syndrome, no proper management has been described. Moreover, the risk of reoccurrence as well as the possibility of malignant transformation into syringofibrocarcinoma (1 case described with Clouston syndrome) is unknown.^6^ In this report, we present an initial management strategy for ESFA using ablative laser.

## Case report

**Fig 1:**
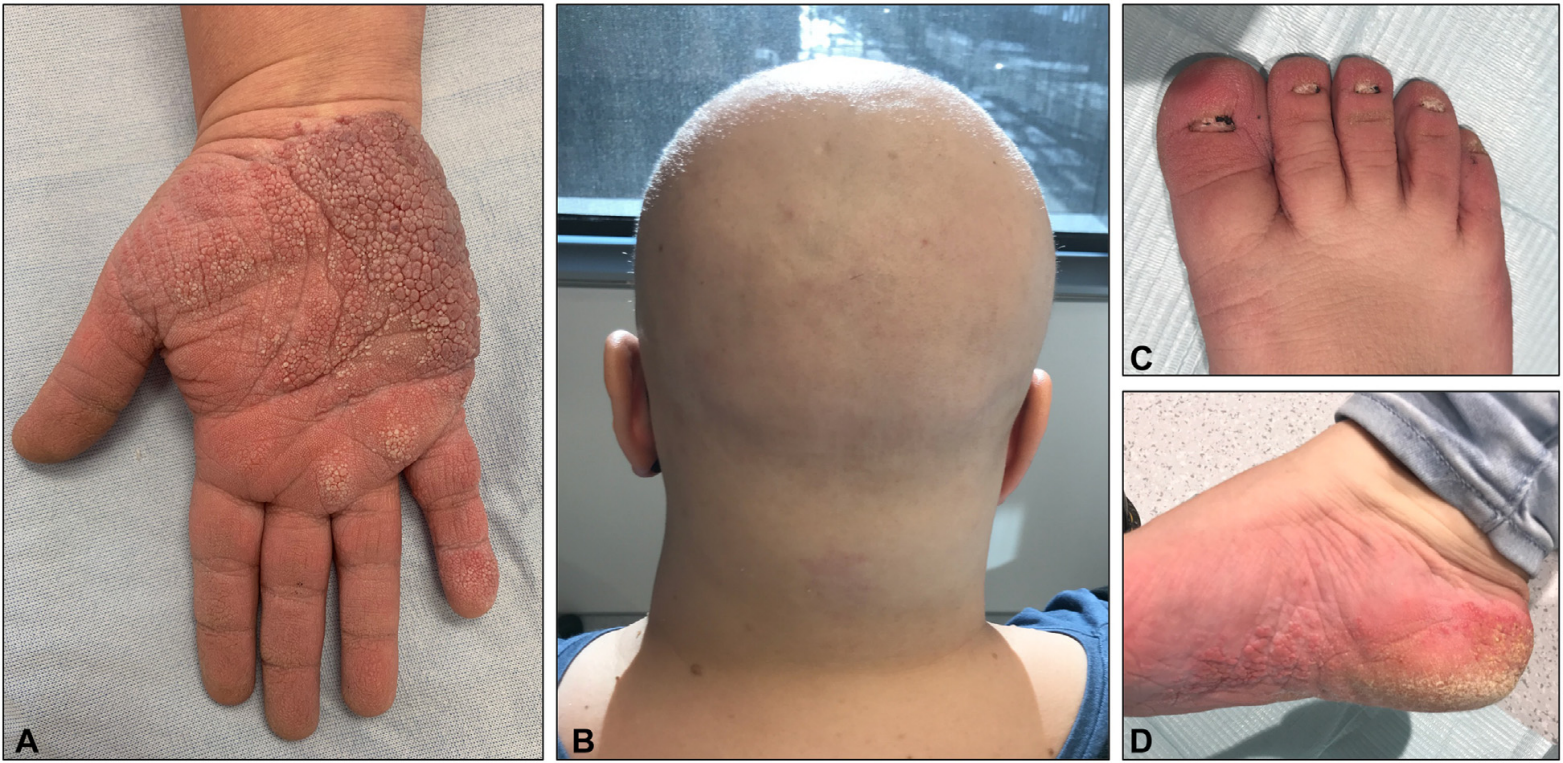
Clinical characteristics of the patient suffering from eccrine syringofibroadenoma associated with Clouston syndrome and presenting with (**A**) *pink* palmar polygonal coalescent papules, (**B**) noncicatricial alopecia, (**C**) onychodystrophy, and (**D**) plantar keratoderma.

**Fig 2:**
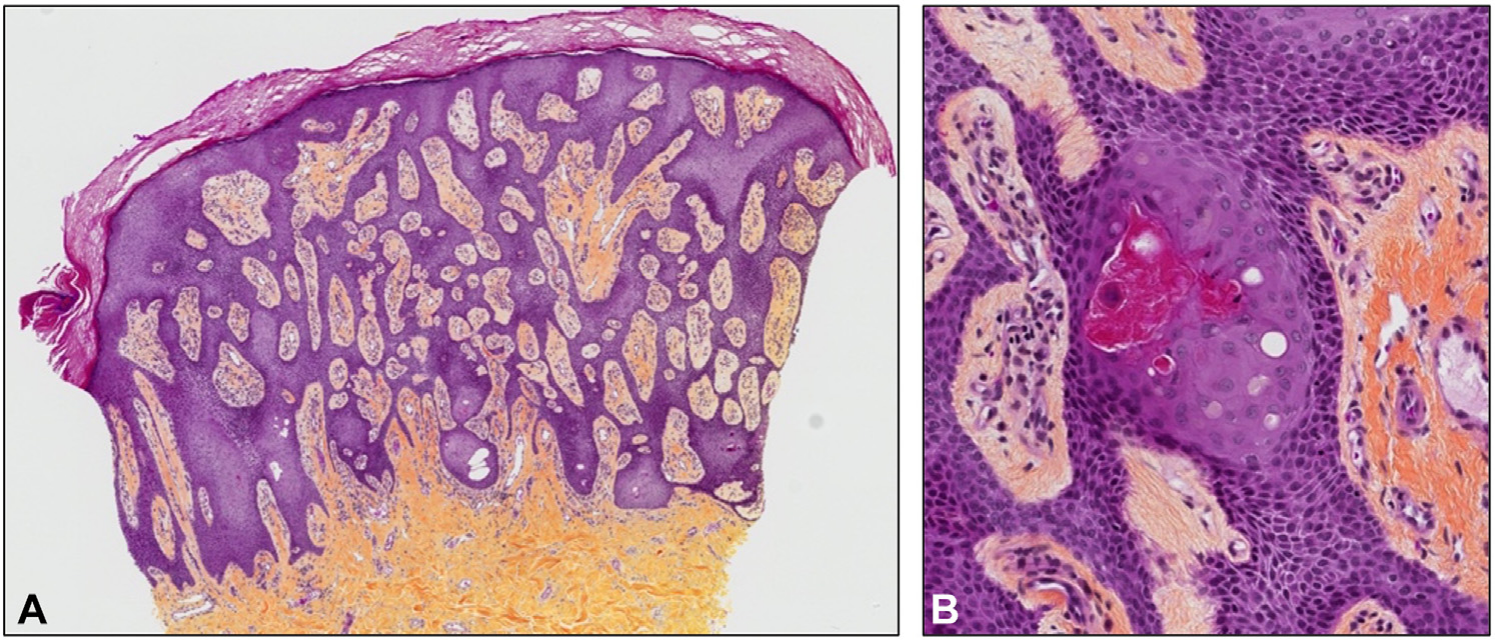
Histology of a palmar eccrine syringofibroadenoma stained with hematoxylin phloxine saffron in a patient with Clouston syndrome. **A,** At low magnification, strands composed of monomorphous epithelial cells are found to be arranged in a reticular fashion extending from the epidermis amid a fibrovascular stroma. **B,** At high magnification, ductal differentiation is observed.

**Fig 3:**
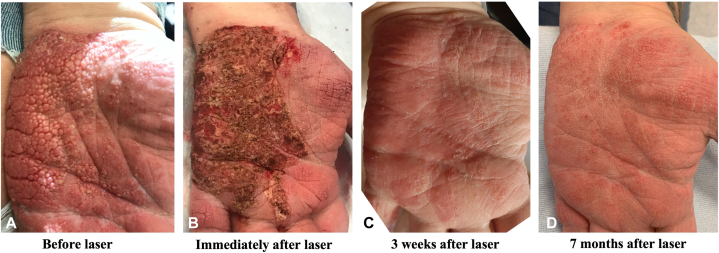
Eccrine syringofibroadenoma of the left hand of the patient (**A**) before, (**B**) immediately, (**C**) 3 weeks; and (**D**) 7 months after the CO_2_ ablative laser.

A 36-year-old French-Canadian woman presented with a history of progressive bilateral palmar lesions (Fig 1, *A*), evolving for the past 10 years, associated with pain secondary to fissures and mild occasional bleeding for which 1 cycle of cryotherapy was unsuccessfully attempted. The patient had a known history of Clouston syndrome and was born with congenital alopecia (Fig 1, *B*) and dystrophic nails (Fig 1, *C*) and later developed palmoplantar keratoderma (Fig 1, *A*, *D*). Genetic testing for ectodermal dysplasia confirmed that the patient had the mutation gap junction protein beta-6 gene c.31G>A, p.(Gly11Arg). Moreover, the 62-year-old mother of the patient was known to have Clouston syndrome and presented with similar palmar lesions, although they were less extensive. On physical examination of the 36-year-old patient, pink erythematous polygonal coalescent papules forming verrucous plaques were observed on the hypothenar regions of the bilateral hyperkeratotic palmar surfaces (Fig 1, *A*). The plantar surfaces were also affected (Fig 1, *D*). Biopsy was compatible with the diagnosis of ESFA. Histologically, large cords with a reticular pattern of monomorphous cells extending from the epidermis into the dermis were observed. There were small ductal structures with a round lumen and an eosinophilic cuticle (Fig 2). Considering the localization and extent of the lesions and their functional impact, CO_2_ ablative laser was recommended to the patient for her nondominant left hand. A wrist nerve block was performed along with local palmar anesthesia using lidocaine 2%. We shaved a portion of the palmar surface of the affected left hand, followed by electrodessication. Additionally, after removing the damaged skin with a gauze, ablative therapy was attempted on the same areas immediately to achieve a long-lasting result (Fig 3) using the CO_2_RE laser (Candela Medical). Treatment was performed with one pass using a spot size of 6 × 6 mm and a fluence of 56.4 J/cm^2^.

Standard dressings were used for the postoperative period, ie, petroleum jelly, followed by sterile dry gauzes, was applied on the wound, and the dressings were secured on the hand using a Kling rolled gauze. The dressing was changed by the patient herself every day after cleaning the area with water until proper healing was achieved. The next 3 days following the laser procedure, the patient experienced mild pain, which was managed with acetaminophen, and the pain gradually subsided. The treated area recovered rapidly within a few days and complete healing was observed at 3 weeks (Fig 3, *C*). During the follow-up period, the patient was significantly satisfied and reported an increased use of her left hand, stating better functionality and enhanced self-confidence associated with the improved esthetic appearance of her hand. Seven months after the laser treatment, the results remain highly satisfying with minimal evidence of ESFA regrowth in the treated area (Fig 3, *D*).

## Discussion

No treatment has been described for the specific case of ESFA occurring with Clouston syndrome. However, some treatments have been tried in other types of ESFA with variable degrees of success, namely surgical excision (if solitary lesion), cryotherapy, radiotherapy, photodynamic therapy, or systemic retinoids.^7^, ^8^, ^9^ Although there are a few reports of ESFA successfully treated with CO_2_ ablative laser,^5^ to our knowledge, there is no published case of CO_2_ ablative laser treatment in an ESFA associated with Clouston syndrome. Our patient was treated with CO_2_ ablative laser, which led to regained functionality of her treated hand and satisfying esthetic results without complications. CO_2_ ablative laser offers a good therapeutic option with short recovery period and sustained results. However, the risks of recurrence and scarring need to be considered along with proper follow-ups in the rare event of a carcinomatous transformation.^6^

## Conflicts of interest

None disclosed.
